# Thoracoscopic Management of Recurrent Pneumothorax in an Adult Patient with Cri du Chat Syndrome and Severe Scoliosis: A Case Report

**DOI:** 10.70352/scrj.cr.26-0421

**Published:** 2026-10-10

**Authors:** Hiroki Ebana, Jun Naito, Yuki Onozato, Takahide Toyoda, Aki Kobayashi, Mizuto Otsuji

**Affiliations:** Department of Thoracic Surgery, Tokyo Metropolitan Bokutoh Hospital, Tokyo, Japan

**Keywords:** cri du chat syndrome, recurrent pneumothorax, severe scoliosis, thoracic deformity, thoracoscopic surgery

## Abstract

**INTRODUCTION:**

Cri du chat syndrome (CdCS) is a rare genetic disorder that can be associated with scoliosis and marked thoracic deformity. Although scoliosis has been reported to be associated with an increased incidence of pneumothorax, reports of pneumothorax surgery in patients with severe scoliosis associated with CdCS are extremely rare. Herein, we report a case of recurrent pneumothorax in an adult patient with CdCS associated with severe scoliosis who was successfully treated by thoracoscopic surgery.

**CASE PRESENTATION:**

A 41-year-old man with CdCS presented with severe scoliosis, intellectual disability, and dental abnormalities. He was transported to our emergency department with severe left pneumothorax associated with complete left lung collapse and marked hypoxemia. CT after chest drainage revealed a bulla located in the deep thoracic cavity near the mediastinum. Because severe thoracic deformity raised concerns regarding both airway management and thoracoscopic manipulation, the patient was initially managed conservatively. However, severe left pneumothorax repeatedly recurred, and surgical intervention was ultimately indicated. Thoracoscopic surgery was performed under 1-lung ventilation using a bronchial blocker. Intrathoracic manipulation was technically challenging because of the marked thoracic deformity associated with severe scoliosis; however, safe operative exposure was achieved using same-axis thoracoscopic manipulation with the thoracoscope and instruments through the same port. A broad-based causative bulla located near the S6–S10 boundary adjacent to the descending thoracic aorta was ligated using ENDOLOOP, followed by intrathoracic administration of 20 mL of 50% glucose solution. The postoperative course was uneventful with no recurrence during 7 years of follow-up.

**Conclusions:**

In patients with severe scoliosis associated with CdCS, thoracic deformity may complicate both airway management and thoracoscopic manipulation. However, thoracoscopic surgery may be feasible with appropriate anesthetic planning and adaptation of the operative strategy. In particular, same-axis thoracoscopic manipulation using the thoracoscope and instruments through the same port may be useful for maintaining safe visualization and instrument handling within a narrow thoracic cavity.

## Abbreviations

CdCS
: cri du chat syndrome
VATS
: video-assisted thoracoscopic surgery

## INTRODUCTION

CdCS is a rare genetic disorder caused by deletion of the short arm of chromosome 5 (5p−), with an estimated incidence of approximately 1 in 50000 live births.^1,2)^ Major clinical features of CdCS include the characteristic cat-like cry, intellectual disability, dental abnormalities, epilepsy, and scoliosis.^1,2)^ Scoliosis is particularly common in patients with CdCS and may result in marked thoracic deformity and narrowing of the thoracic cavity.^3)^ In addition, scoliosis has been associated with an increased incidence of spontaneous pneumothorax, and altered intrathoracic mechanics caused by thoracic deformity may contribute to its development.^4,5)^

However, reports of thoracoscopic surgery for recurrent pneumothorax in patients with severe scoliosis associated with CdCS are extremely rare. Furthermore, in patients with marked thoracic deformity, thoracoscopic surgery can be technically challenging because of both difficult lung separation associated with airway deviation and restricted intrathoracic manipulation within a narrow thoracic cavity.

Herein, we report a case of recurrent pneumothorax in an adult patient with CdCS associated with severe scoliosis and marked thoracic deformity who was successfully treated by thoracoscopic surgery. In this case, safe surgical manipulation was achieved using same-axis thoracoscopic manipulation based on the Uniportal VATS concept combined with a flexible reduced-port strategy.

This case report was prepared in accordance with the CARE guidelines.

## CASE PRESENTATION

A 41-year-old man who had been diagnosed with CdCS in infancy presented with severe scoliosis (Cobb angle, 116°), dental abnormalities, and intellectual disability. According to his medical records, a chromosomal abnormality was identified at approximately 3 months of age, and a 5p deletion was documented in his subsequent medical history. However, the original cytogenetic report and the specific testing method were not available for review. He had a small body habitus, with a height of 137 cm and a body weight of 29 kg and showed marked thoracic deformity. He was transported to our emergency department because of abnormal behavior. On arrival, his blood pressure was 141/83 mmHg, heart rate was 128 beats/min, respiratory rate was 20 breaths/min, and oxygen saturation was 82% on room air. Chest radiography demonstrated complete collapse of the left lung (**Fig. 1A**). Because of his intellectual disability, a chest tube was inserted under sedation, resulting in improvement of oxygen saturation to 100% with oxygen at 5 L/min via face mask. Chest CT performed after drainage revealed a deep bulla near the mediastinum (**Fig. 1B**), as well as marked thoracic deformity and narrowing of the thoracic cavity associated with severe scoliosis (**Fig. 1C**, **1D**).

**Fig. 1:**
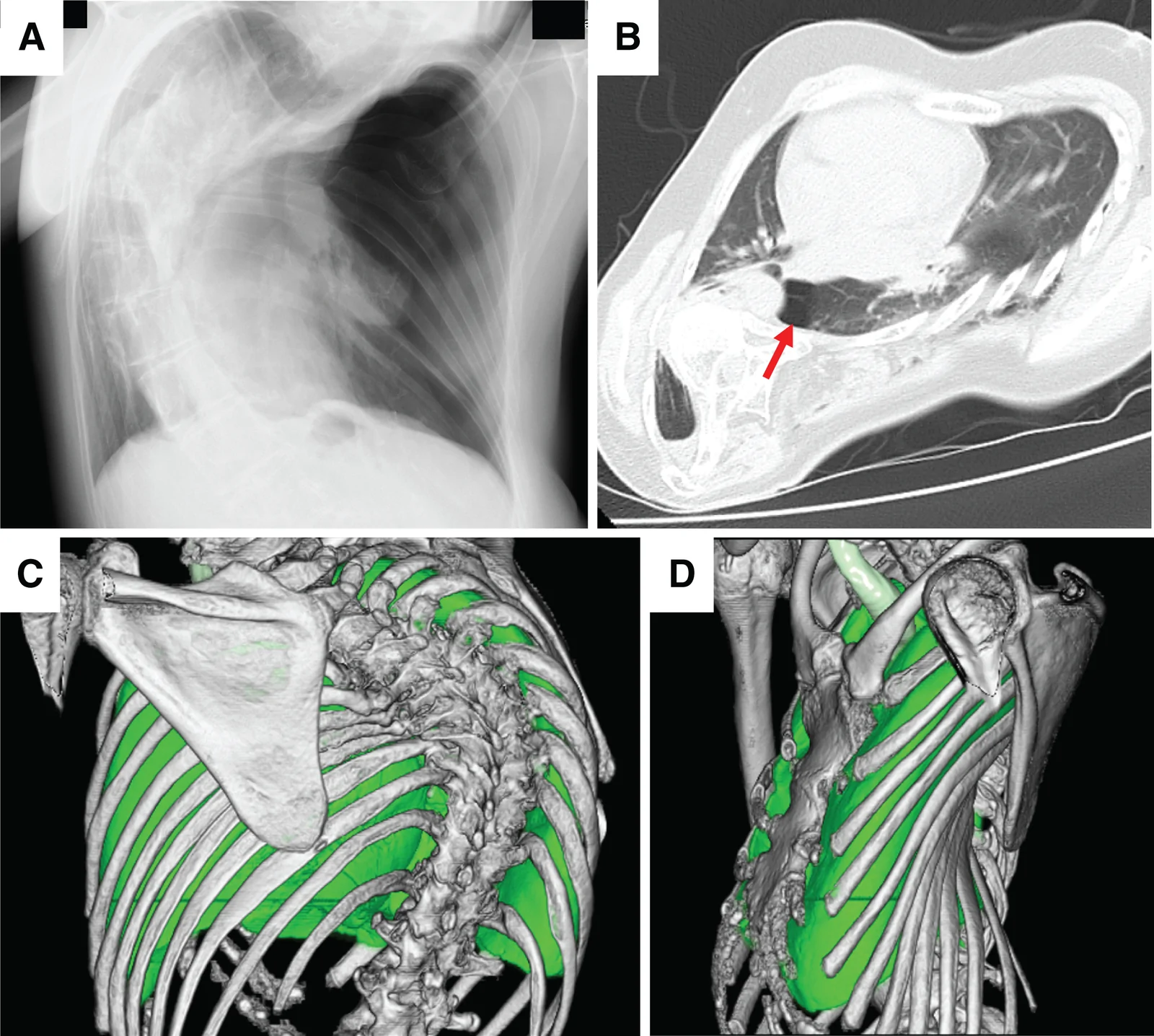
Preoperative imaging findings. (**A**) Chest X-ray showed severe scoliosis and complete collapse of the left lung due to pneumothorax. (**B**) Chest CT after drainage demonstrated a deep bulla near the mediastinum (arrow). (**C**, **D**) 3D CT demonstrated severe scoliosis, marked thoracic deformity, and narrowing of the thoracic cavity.

After chest drainage, the air leak resolved promptly, and the chest tube was subsequently removed. Although surgical treatment was considered, it was initially deferred because a severe thoracic deformity, airway deviation, and an intellectual disability raised substantial concerns regarding general anesthesia, 1-lung ventilation, and thoracoscopic manipulation. He subsequently experienced 3 additional episodes of severe left pneumothorax, each associated with the complete collapse of the left lung and a decrease in oxygen saturation to the 80% range. The air leak again resolved promptly after chest drainage during each episode. After the third episode, surgical treatment was considered; however, general anesthesia and 1-lung ventilation were considered technically challenging at our institution. Therefore, a multidisciplinary plan was established to perform surgery with the assistance of a pediatric anesthesiologist experienced in managing patients with complex congenital disorders in the event of a recurrence. When the fourth episode occurred, surgical intervention was performed according to this plan.

General anesthesia was induced using an 8.0-mm single-lumen endotracheal tube. A 3.0-mm COOPDECH Endobronchial Blocker Tube (Daiken Medical, Osaka, Japan) was placed in the left main bronchus, and its position was confirmed by fiberoptic bronchoscopy. One-lung ventilation was successfully established. The patient was placed in the right lateral decubitus position; however, severe scoliosis and marked thoracic deformity resulted in an unusual body position (**Fig. 2A**). Thoracoscopic observation was first performed using a uniportal approach through the previous chest tube insertion site. Because of the severe thoracic deformity, the intrathoracic space was markedly narrowed. By inserting both the thoracoscope and instruments through the same port and performing same-axis thoracoscopic manipulation, the causative bulla located deep within the thoracic cavity was identified (**Fig. 2B**).

**Fig. 2:**
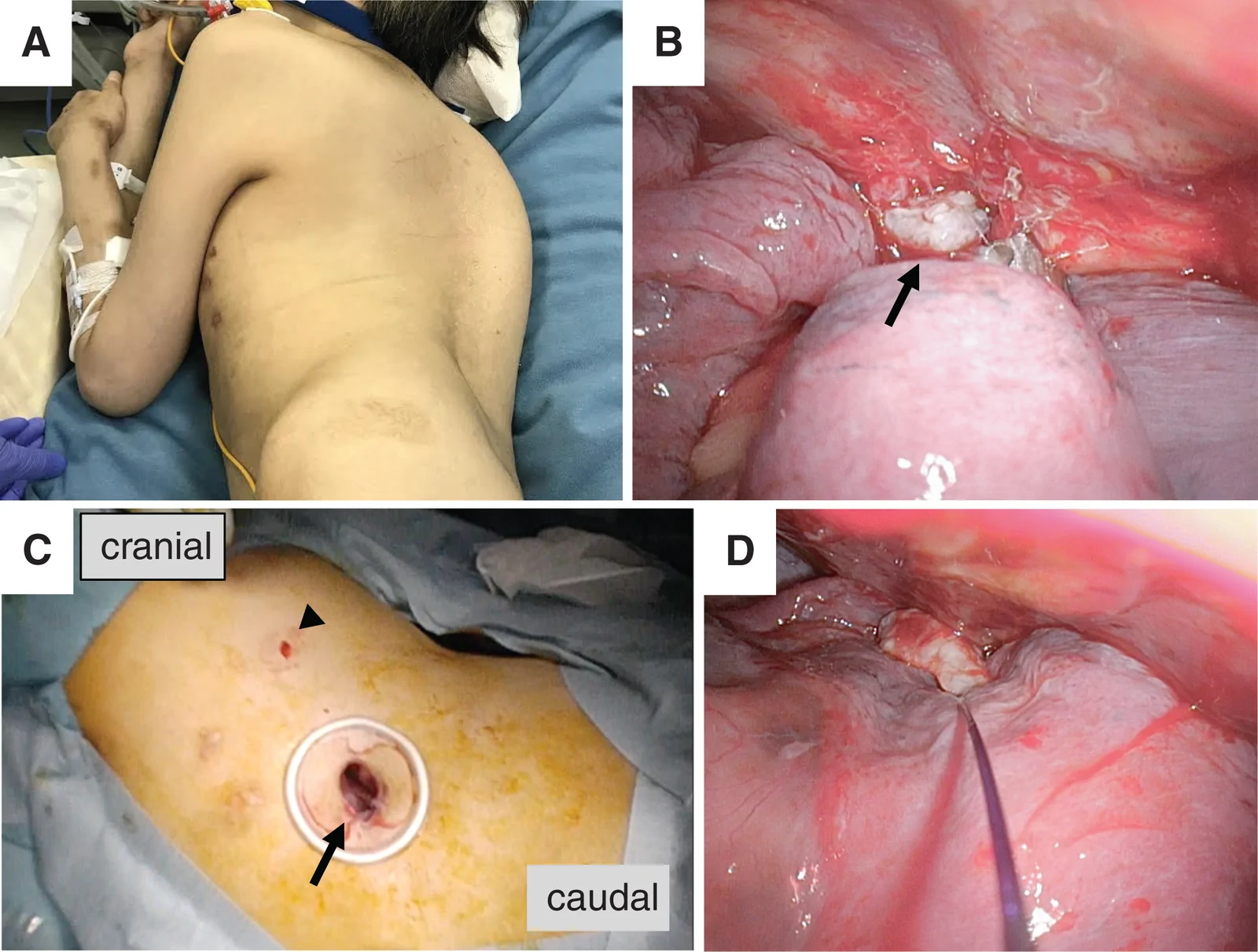
Operative findings. (**A**) The patient presented with marked thoracic deformity due to severe scoliosis associated with CdCS. (**B**) Thoracoscopic view showing the causative bulla located in the deep thoracic cavity near the mediastinum (arrow). (**C**) Intraoperative external view. A wound retractor was inserted through the main incision (arrow), and an additional 7-mm port was created within the same intercostal space (arrowhead), enabling aligned camera–instrument axis manipulation. (**D**) The causative bulla was ligated using ENDOLOOP (Ethicon, Johnson & Johnson, New Brunswick, NJ, USA). CdCS, cri du chat syndrome

The procedure was initially started using a uniportal approach to maximize camera–instrument alignment within the extremely narrow thoracic cavity. However, because the thoracic cavity was extremely narrow and strong cardiac pulsation further restricted the operative field, stable operative exposure and safe manipulation could not be consistently maintained through the Uniportal approach. Therefore, an additional 7-mm port was created, and the procedure was converted to 2-port VATS (**Fig. 2C**, **Video 1**).

A retraction instrument was inserted through 1 port, while the thoracoscope and operative instruments were inserted through the other port, enabling safe visualization and manipulation even within the narrow thoracic cavity. The causative bulla was located near the descending thoracic aorta at the boundary between the superior segment (S6) and posterior basal segment (S10) of the left lower lobe and showed a broad base with wall thickening. Because severe thoracic deformity and the deep mediastinal location of the lesion prevented a safe insertion angle for an endoscopic stapler, ligation using ENDOLOOP (Ethicon, Johnson & Johnson, New Brunswick, NJ, USA) was selected instead of stapled resection. Subsequently, 20 mL of 50% glucose solution was sprayed into the thoracic cavity as a pleural-coating procedure to reduce the risk of postoperative pneumothorax recurrence^6)^ (**Fig. 2D**). Operative time was 50 minutes, and blood loss was 10 g.

The postoperative course was uneventful. The chest tube was removed on POD 1, and the patient was discharged home on POD 6. No recurrence has been observed during 7 years of follow-up after surgery.

## DISCUSSION

This case has 2 important clinical implications. First, recurrent pneumothorax can occur in patients with severe scoliosis associated with CdCS. Second, even in patients with marked thoracic deformity, thoracoscopic surgery may be feasible when the operative strategy is adapted according to the thoracic geometry.

Takebayashi et al. reported scoliosis in 8 of 11 patients (73%) with CdCS and suggested that abnormal muscle tone may contribute to scoliosis progression.^3)^ However, reports of pneumothorax surgery in patients with severe scoliosis associated with CdCS are extremely rare. Pneumothorax associated with scoliosis has also been reported in connective tissue disorders such as Marfan syndrome.^7)^ Furthermore, scoliosis itself has been associated with an increased incidence of spontaneous pneumothorax.^4)^ Thoracic deformity may alter intrathoracic pressure distribution and increase mechanical stress on the lung apex, thereby contributing to bulla formation and pneumothorax development.^5)^ In the present case, severe scoliosis and thoracic deformity may have altered intrathoracic mechanics; however, a direct causal relationship with bulla formation and recurrent pneumothorax cannot be established. The markedly low BMI (15.5 kg/m^2^) may also have contributed, suggesting that the development of pneumothorax in this patient was likely multifactorial.

Thoracoscopic surgery in patients with severe scoliosis presents 2 major challenges: airway management and intrathoracic manipulation. Severe thoracic deformity and airway deviation may complicate 1-lung ventilation and lung separation.^8,9)^ In the present case, safe operative exposure was achieved using a bronchial blocker. The “airway first” concept, in which secure airway establishment is prioritized before lung separation, has been emphasized in patients with anticipated difficult airways,^8,9)^ and this strategy was considered useful in the present case.

Severe thoracic deformity also causes marked narrowing and asymmetry of the thoracic cavity, making conventional triangulation-based multiportal VATS difficult. In patients such as the present case, where adequate distance between ports cannot be secured, instrument interference and restricted maneuverability may impair safe thoracoscopic manipulation.

In the present case, both the thoracoscope and instruments were initially inserted through the same port. After confirming that the instrument had been safely introduced into the thoracic cavity, the thoracoscope was inserted along the same axis to establish same-axis thoracoscopic manipulation. This strategy enabled all instruments to remain under direct visualization even within the narrow thoracic cavity, thereby reducing the risk of inadvertent lung injury. Furthermore, the additional port was placed within the same intercostal space as the main incision, and the camera axis was aligned with the main wound, allowing maintenance of a surgical view and operative axis similar to those of Uniportal VATS even after conversion to 2-port VATS. Because the visual axis and instrument direction were aligned in the same plane, this approach was advantageous for accessing deep intrathoracic lesions.^10)^ Same-axis thoracoscopic manipulation itself is not a novel geometric concept, but rather represents an established principle of Uniportal VATS. The distinctive feature of the present case was the application of this principle to an extremely narrow and severely distorted thoracic cavity and the flexible transition from a Uniportal to a 2-port approach while preserving camera–instrument alignment. Although stapled resection is generally preferred in pneumothorax surgery, ligation may represent a practical alternative when thoracic geometry prevents safe stapler deployment.

Uniportal VATS has become widely accepted as a minimally invasive thoracic surgical approach.^11–13)^ Its advantages include not only reduced postoperative pain and improved cosmesis but also reduced instrument interference within narrow thoracic cavities. Hsu et al. demonstrated that uniportal thoracoscopic surgery could be safely performed even in patients with severe thoracic scoliosis by adapting port placement and operative axis design according to thoracic geometry.^11)^ In patients with severe thoracic deformity such as the present case, same-axis thoracoscopic manipulation may therefore provide advantages over conventional triangulation-based approaches.

## CONCLUSIONS

We successfully performed thoracoscopic surgery for recurrent pneumothorax in an adult patient with CdCS associated with severe scoliosis and marked thoracic deformity. In patients with severe scoliosis, both airway management and intrathoracic manipulation can be challenging; however, the combination of lung separation using a bronchial blocker and same-axis thoracoscopic manipulation enabled safe operative exposure and instrument handling even within a narrow thoracic cavity. Furthermore, flexible adaptation of the operative strategy according to thoracic geometry, including placement of an additional port within the same intercostal space, facilitated safe completion of the procedure. This approach may represent a practical minimally invasive thoracoscopic strategy for patients with severe thoracic deformity and restricted intrathoracic working space.

## SUPPLEMENTARY MATERIALS

Video 1 Thoracoscopic surgery for recurrent pneumothorax in a patient with cri du chat syndrome associated with severe scoliosis and marked thoracic deformity. The thoracic cavity was extremely narrow because of the severe thoracic deformity, and strong cardiac pulsation in the left thoracic cavity limited the operative field. Thoracoscopic manipulation was initially performed through a uniportal approach with alignment of the camera and instrument axes, followed by conversion to 2-port VATS with placement of an additional port within the same intercostal space to maintain safe exposure and manipulation. A broad-based bulla located near the S6–S10 boundary adjacent to the descending thoracic aorta was ligated using an ENDOLOOP instead of stapled resection because safe stapler deployment was difficult in the narrow thoracic cavity.

## DECLARATIONS

### Availability of data and materials

All data generated or analyzed during this study are included in this published article.
